# *In utero* Exposure to Valproic-Acid Alters Circadian Organisation and Clock-Gene Expression: Implications for Autism Spectrum Disorders

**DOI:** 10.3389/fnbeh.2021.711549

**Published:** 2021-09-28

**Authors:** Sarah Ferraro, Nuria de Zavalia, Nicolas Belforte, Shimon Amir

**Affiliations:** ^1^Department of Psychology, Center for Studies in Behavioural Neurobiology, Concordia University, Montreal, QC, Canada; ^2^Department of Neuroscience, University of Montreal Hospital Research Center, Montreal, QC, Canada

**Keywords:** autism spectrum disorder, circadian rhythms, valproic acid, clock-gene, rodent model, rhythm disturbances

## Abstract

Autism Spectrum Disorder (ASD) is a pervasive neurodevelopmental disorder characterised by restrictive patterns of behaviour and alterations in social interaction and communication. Up to 80% of children with ASD exhibit sleep-wake cycle disturbances, emphasising the pressing need for novel approaches in the treatment of ASD-associated comorbidities. While sleep disturbances have been identified in ASD individuals, little has been done to assess the contribution of the circadian system to these findings. The objective of this study is to characterise circadian behaviour and clock-gene expression in a valproic acid (VPA)-induced animal model of autism to highlight perturbations potentially contributing to these disturbances. Male and female VPA-exposed offspring underwent circadian challenges, including baseline light-dark cycles, constant dark/light and light pulse protocols. Baseline analysis showed that VPA-exposed males, but not females, had a greater distribution of wheel-running behaviour across light-dark phases and a later activity offset (*p* < 0.0001), while controls showed greater activity confinement to the dark phase (*p* = 0.0256). Constant light analysis indicated an attenuated masking response and an increase in the number of days to reach arrhythmicity (*p* < 0.0001). A 1-h light pulse (150 lux) at CT 15 after 6 days of constant dark showed that both sexes exposed to VPA exhibited a lesser phase-shift when compared to controls (*p* = 0.0043). Immunohistochemical and western-blot assays reveal no alterations in retinal organisation or function. However, immunohistochemical assay of the SCN revealed altered expression of BMAL1 expression in VPA-exposed males (*p* = 0.0016), and in females (*p* = 0.0053). These findings suggest alterations within the core clockwork of the SCN and reduced photic-entrainment capacity, independent of retinal dysfunction. The results of this study shed light on the nature of circadian dysregulation in VPA-exposed animals and highlights the urgent need for novel perspectives in the treatment of ASD-associated comorbidities.

## Introduction

Autism Spectrum Disorder (ASD) is a pervasive neurodevelopmental disorder affecting ~1–2% of individuals in Western societies, where males are disproportionately diagnosed four times more than females (Maenner et al., 2020). ASD is characterised by restrictive and repetitive patterns of behaviour and variances in social communication and reciprocity, though the aetiology and development of the disorder remain unclear (American Psychiatric Association, 2013). Individuals with ASD often present concomitantly with disorders such as epilepsy, depression, attention deficit-hyperactivity disorder and anxiety (Crawley, 2012; Varghese et al., 2017). However, among the most frequently observed comorbidities is that of sleep-wake cycle disturbances, affecting up to 80% of children with ASD (Richdale, 1999; Glickman, 2010). Most often, these alterations manifest as increased sleep latencies, waking during the night and difficulty arising in the morning (Richdale and Prior, 1995; Wiggs, 2004; Baker and Richdale, 2015). Additionally, evidence points to abnormal temporal secretion profiles of cortisol and melatonin in children with ASD, collectively suggesting an underlying impairment of the circadian timing system (Richdale and Prior, 1992; Corbett et al., 2008).

In mammals, circadian rhythms are controlled by a distributed network of central and peripheral clocks governed by a master pacemaker located in the suprachiasmatic nucleus (SCN) of the hypothalamus (Rosbash, 1995; Takahashi et al., 2008). These cellular clocks anticipate and prepare physiological and behavioural processes to align with predictable environmental changes, namely that of the earth's 24-h light-dark (LD) cycle. This entrainment to solar time results from direct innervation of the SCN by intrinsically photosensitive retinal ganglion cells (ipRGC's) that express the photopigment melanopsin (Berson et al., 2002). Entrainment to the light-dark cycle and synchronisation of peripheral clocks by the SCN is necessary for physiological and psychological well-being; thus, it comes as little surprise that disruption within this system has been associated with a host of pathologies, including cancer, diabetes, and affective disorders (Takahashi et al., 2008; Logan and McClung, 2019). At the cellular level, an evolutionarily conserved circadian timekeeping mechanism within the SCN and peripheral tissues begins with self-sustaining, autoregulatory transcriptional-translational feedback loops (Hastings et al., 2018). Within the core loop, the CLOCK/BMAL1 transcription factors are involved in the rhythmic transcription of their own inhibitors, *Period* (*Per1, Per2*) and *Cryptochrome* (*Cry1, Cry2*), whose protein products act to supress CLOCK/BMAL1 transcriptional activity (Takahashi, 2017; Hastings et al., 2018). Ubiquitination and subsequent degradation of the PER/CRY complexes abrogates the inhibitory effect on CLOCK/BMAL1, allowing the cycle to begin anew (Partch et al., 2006; Gallego and Virshup, 2007).

Circadian disturbances in ASD individuals have been reported to emerge in infancy, long before a diagnosis has been made, and persist throughout adolescence and adulthood, potentially presenting lifelong detrimental consequences on physiological and psychological functioning (Baker and Richdale, 2015; Karaivazoglou and Assimakopoulos, 2018). In specific, alterations in sleep and circadian rhythms have been associated with worsened daytime behaviour, decreased seizure threshold, and an overall decrease in quality of life in children with ASD (Richdale and Schreck, 2009; Cohen et al., 2014). As such, sleep and circadian disorders are not solely an adverse consequence of ASD, but ones that can be targeted for future clinical interventions. Thus, identification of the molecular abnormalities underlying circadian dysregulation in ASD would allow for the development of targeted strategies for reinstating rhythmicity and its associated behaviours. However, research evaluating the circadian system as a contributing factor in diurnal alterations in ASD individuals remains sparse.

To gain insight into these questions, we utilised a valproate (VPA)-animal model of ASD. While VPA is frequently prescribed as an anticonvulsant and mood stabilising medication, it is also a known human teratogen with well-documented links to the development of ASD after *in utero* exposure (Clayton-Smith and Donnai, 1995). Administration of VPA on gestational day 12.5 in rodents results in pups that display behavioural and neuroanatomical characteristics akin to what is seen in human ASD populations, including decreased social engagement, hypo-and-hypersensitivity to sensory stimuli and increased stereotypic behaviours (Rodier et al., 1996; Schneider and Przewłocki, 2005). Moreover, male rodents are found to exhibit decreased sociability more frequently than their female littermates, recapitulating the sex differences seen in human ASD populations (Schneider et al., 2008; Kataoka et al., 2013). As such, the VPA model offers an excellent opportunity to study circadian disturbances in ASD. The objective of this study is to functionally describe circadian behaviour and clock-gene expression in a VPA-induced model of ASD to highlight perturbations potentially contributing to this well-known ASD-comorbid phenotype.

## Methods and Materials

### Animals and Housing

All experimental procedures followed the guidelines set by the Canadian Council on Animal Care and approved by the Animal Care Committee of Concordia University. Time-pregnant female Wistars (4 months of age) were purchased from Charles River Canada (Charles River, St-Constant, QC) and were delivered on gestational day (GD) ten. Dams were individually housed in clear plastic cages with *ad libitum* access to food and water and kept under a 12 h light-dark (LD) cycle in the animal care facility. On GD 12, dams received an intraperitoneal injection of either VPA dissolved in saline (500, 1 mg/kg) or 0.9% saline solution (1 mg/kg). The resulting litters were not culled, and mothers tended to their pups with minimal interference. Pups were subjected to behavioural assays to measure developmental markers between PND 2 and 10. Weaning occurred on PND 23.

Between PND 24 and 30, pups were introduced to sound-attenuated isolation boxes equipped with running wheels and *ad libitum* access to food and water. Wheel-running activity was collected continuously by VitalView software (Mini-Mitter, Starr Life Sciences Corp., Oakmont, PA, USA). Graphical representation of wheel-running behaviour was acquired by using Actiview Biological Rhythm Analysis software (Mini-Mitter) and analysed using Clocklab (Actimetrics, Evanston, IL, USA).

### Developmental Assessments

#### Righting Reflex Task

On PND 2–5 male pups were placed in a supine position on a table. The time to fully right themselves by 180° was recorded, up to a 30-s maximum. Promptly after assessment, pups were reintroduced to their home cages to avoid undue stress.

#### Olfactory Discrimination Task

Between PND 9 and 10 male and female pups were placed in the centre of a clean, transparent standard housing cage with two olfactory stimuli at opposite ends of the cage. The stimuli used were clean SaniChip bedding (neutral odour) and home cage bedding (nest odour) and were placed in two separate weighing boats. Latency to reach and engage with the home bedding was recorded up to a 3-min maximum. The cage was sanitised with a 70% ethanol solution between subjects.

### Circadian Analysis

Initially, male, and female offspring were placed in a standard 12 h LD cycle (150 lux) for 2 weeks to determine the following parameters of the behavioural rhythm: activity onset/offset, amplitude, alpha, intra-daily variability (a quantification of rhythm fragmentation), and inter-daily stability (a quantification of the synchronisation strength between the endogenous rhythm and the external LD cycle). Importantly, activity events during the light and dark phases were determined by activity bout analysis with a defined threshold (1-wheel revolution/min/10 min). Notably, the last 7 days of the 2-week 12 h LD cycle were used to determine these parameters and calculated by Clocklab software (Actimetrics, Evanston, IL, USA) analysis tool. Animals were then placed in constant dark (DD) for 3 weeks and the endogenous free-running period (tau) of each animal was determined. During this time, a 1-h 150 lux light pulse (LP) was administered 3 h after the onset of running-wheel activity (CT 15). CT 15 was determined for each animal on the day of the LP by fitting a regression line to the actogram obtained from wheel running. The subsequent phase shift (in hours) was calculated by subtracting the predicted regression line from the regression line post-LP and averaged over 5 days. Finally, a constant light (LL, 150 lux) protocol was employed for 1 month before the termination of the experiment. The number of days from the onset of LL to the emergence of arrhythmic behaviour was documented, initially through visual inspection of the actogram by three independent raters blind to the experimental conditions. These findings were also corroborated by fitting a regression line to the onset of activity during the first 7 days of LL and documenting the day in which a clear activity onset could no longer be observed and was discrepant with the predicted onset determined by the linear regression. Masking behaviour was calculated by averaging total activity for 7 days in 12 h LD/total activity for the first 7 days in LL. Results were obtained through use of the Clocklab software (Actimetrics, Evanston, IL, USA) analysis tool.

### Tissue Preparation and Immunohistochemistry

To discern rhythmic expression differences between groups, four time points for tissue harvesting were selected [Zeitgeber time (ZT) 1, 7, 13, and 19, where ZT 0 denotes time of lights on and ZT 12 denotes time of lights off. Typically, *n* = 3 animals/time point]. Fourteen to eighteen weeks old rats were anaesthetised with sodium pentobarbital (100 mg/kg, intraperitoneally) and transcardially perfused with 300 ml of cold saline (0.9% NaCl), followed by 300 ml of cold paraformaldehyde (4% in a 0.1M phosphate buffer, pH 7.3). Brains were extracted and post-fixed for 24 h in paraformaldehyde at 4°C, then sliced coronally at 30 or 50 μm for immunofluorescence or immunohistochemistry assays, respectively, and stored at −20°C in Watson's Cryoprotectant.

Sections containing the SCN were identified and immunohistochemically stained for either the circadian clock-gene, BMAL1 (1:30,000; made in rabbit, polyclonal, Novus Biologicals, NB100-2288), or p75NTR, a marker of retinal connectivity (1:8,000; made in rabbit, polyclonal, Millipore Sigma #07-476). The use, antibody specificity and quantification of BMAL1 has previously been described by others (Gall et al., 2003; Wyse and Coogan, 2010; Al-Safadi et al., 2014; de Zavalia et al., 2021). In brief, the primary antibody solution was made in a milk buffered (5%) triton-TBS solution and 2% normal goat serum and incubated for 48 h at 4°C. After rinsing the free-floating tissue in TBS, a secondary incubation solution containing biotinylated anti-rabbit IgG made in goat (1:200 dilution; Vector Laboratories, Burlington, ON, Canada) was used. Brain sections were rinsed in fresh TBS and then incubated in an avidin-biotin solution for 2 h (4°C; Vectastain Elite ABC Kit; Vector Laboratories). Finally, for visualisation of immunoreactive cells, sections were rinsed in a 0.5% 3,3-diaminobenzidine (DAB) solution (10 min), followed by a solution of 0.5% 3,3-DAB, 0.01% H_2_O_2_, and 8% NiCl_2_ (10 min). Subsequently, sections were mounted onto gel-coated glass slides and underwent serial alcohol dehydration, cleared with Citrisolv (Fisher Scientific, Pittsburgh, PA, USA) and coverslipped. Examination of the sections of interest was conducted *via* the use of a light microscope (Leica, DMR) and images were captured with a Sony XC-77 video camera, usually with a minimum of eight images per brain region captured. Using ImageJ software (http://imagej.nih.gov), a set dimension was chosen (100 × 100) for the brain region of interest and background staining (40 × 40); the density of the background was subtracted from the density of the area of interest to yield our result.

### Immunofluorescence

Fourteen weeks old rats were subjected to a 1-h light pulse (150 lux) at CT 15 and were sacrificed 1 h after the termination of the light pulse. Double labelling was performed for c-FOS (1/800 dilution, PhosphoSolutions, 309-cFOS) and VIP (1/10,000 dilution, Immunostar, 20077). Free-floating sections were rinsed for 10 min in phosphate-buffered saline (PBS, pH 7.4), followed by 3 × 10 min rinses in 0.3% Triton-X in PBS (PBS-TX). Tissue was pre-blocked for 1 h at room temperature with gentle agitation in a solution of PBS-TX with 3% milk powder and 6% normal donkey serum. The tissue was then incubated at room temperature overnight with gentle agitation in the primary antibody solution. The primary antibody solution was composed of the primary antibody diluted in 2% NDS and 3% milk powder buffer. Finally, a secondary antibody solution containing Alexa-488 (1/1,000 dilution, anti-rabbit) and Alexa-594 (1/1,000 dilution, anti-mouse. Life Technologies, Carlsbad, CA, USA) was used for signal amplification. Tissue was rinsed 3 × 10 min in PBS-TX, and a final 10 min in PBS before being mounted onto slides and allowed to dry. Slides were coverslipped using VECTASHIELD® Antifade Mounting Media (Vector Laboratories, Inc., Burlingame, CA, USA) for fluorescence with DAPI (Vector Laboratories, Inc.). Confocal images were taken using the Olympus FV10i automated confocal laser scanning microscope using a 60x objective at the Centre for Microscopy and Cell Imaging at Concordia University, Montreal, Canada. The location of the SCN was determined based on landmarks from “The Rat Brain in Stereotaxic Coordinates” (Paxinos and Watson, 1997). All confocal parameters (capture area, depth, contrast, brightness, etc.) were kept constant and laser intensity was set automatically and then adjusted for each section.

### CORT ELISA

Trunk blood was collected from animals across 24 h (ZT 1, 7, 13, and 19) after a terminal live decapitation in heparinized blood collection tubes (*n* = 3 animals/time-point). Samples were centrifuged for 10 min at 4°C, 13,000 r.p.m and plasma were extracted and stored at −80°C. Plasma CORT levels were assessed using a CORT Enzyme Immunoassay (ELISA) (Thermofisher Scientific) and the assay was run per manufacturers guidelines as previously described (Al-Safadi et al., 2014).

### Western Blot Analysis

Fourteen to sixteen weeks old rats were entrained for 2 weeks to a 12 h LD cycle and were sacrificed shortly after lights on (between ZT 1 and 3, *n* = 2/condition). The eye was isolated and both retinas were dissected out and frozen on dry ice. The tissue was then homogenised (two retinas per tube from the same sample) in 200 μl of lysis buffer [1M Tris-HCL pH 6.8, 10% sodium dodecyl sulphate, 0.1 ml phosphatase inhibitor cocktail 2 (Sigma #P5726, Burlington, MA, USA), 0.1 ml phosphatase inhibitor cocktail 3 (Millipore Sigma, #P0044, Burlington, MA, USA), 1 × protease inhibitor cocktail (Roche, Basel, Switzerland)]. The extracted proteins (100 μg/lane) were electrophoresed into a 10% SDS-PAGE gel and blotted into a nitrocellulose membrane (Bio-Rad, # 1620112, Hercules, CA, USA). The subsequent membrane was blocked with a 5% milk buffer, 0.3% Tween-PBS solution and incubated overnight at 4°C in 0.3% Tween-TBS with the anti-Melanopsin (1:500 dilution) antibody (ThermoFisher, #PA1-780, Waltham, MA, USA) and anti-actin (1/80,000 dilution) antibody (Millipore Sigma, #A5441, Burlington, MA, USA). Finally, the membrane was incubated in 0.3% Tween-PBS, 5% milk buffer solution with the addition of a goat anti-rabbit and anti-mouse IgG horseradish peroxidase-conjugated antibody (1/2,500 dilution; Millipore Sigma, #AP132P). Signal visualisation was achieved *via* Western Lighting Chemiluminescence light-emitting system (PerkinElmer Life Sciences, Waltham, MA, USA). Between each antibody incubation, membranes were washed in three, 10-min intervals in 0.3% Tween-PBS.

### Statistical Analysis

For activity parameters, differences between groups were analysed with unpaired two-tailed *t*-test (actogram analysis) or one-way analysis of variance (ANOVA). Male and female animals were not compared between groups, as the objective of this study was not to describe differences between the sexes post *in utero* exposure to VPA. Rather, we focus our statistics on how males and females exposed to VPA *in utero* differ from their control counterparts respectively. A two-way ANOVA was utilised for analysing BMAL1 expression in our regions of interest and CORT secretion. A significance threshold was set at α = 0.05 using GraphPad Prism 9 (GraphPad software LLC, San Diego, CA, USA). Planned comparisons were conducted with a Bonferroni correction when appropriate. Corrections for unequal variances were used when appropriate and detailed in the figure captions.

## Results

### Development of VPA-Offspring

To investigate the effect of *in utero* VPA-exposure on the development of the resulting offspring, animals were subjected to various screening assays to assess motor development, ASD-like behaviours, and general health. The weight of male offspring shortly after birth (Supplementary Figure 1A), after weaning and into adulthood (Supplementary Figure 1B) were recorded and were indistinguishable between groups. Since VPA had no effect on overall weight gain in males, the weight of female offspring was recorded shortly after birth and weaning equally and revealed no distinguishable differences between groups (Supplementary Figure 1C). A righting reflex task was performed shortly after birth where VPA and saline-exposed male offspring were placed in a supine position and the time to right themselves ventrally was recorded. No significant differences between groups were detected, indicating normal motor development in VPA-exposed male pups (Supplementary Figure 1D).

Nest-seeking responses mediated by the olfactory system are important for the development of social behaviour in rodent pups (Terry and Johanson, 1996). Deficits in nest-seeking behaviour have been documented in VPA-exposed males but not in VPA-exposed females and are indicative of olfactory discrimination deficits later in development (Roullet et al., 2010; Favre et al., 2013). To verify the presence of known ASD-like behaviours in offspring generated in our laboratory, the olfactory discrimination task (ODT) was conducted on PND 9 and is detailed in the methodology. Consistent with previous reports, males exposed to VPA demonstrated a longer latency to engage with home bedding when compared to controls (Supplementary Figure 1E) (*p* < 0.0005). However, this effect was not seen in VPA-exposed females (Supplementary Figure 1F). These results indicate the presence of ASD-like behaviours in offspring while the general health and development of VPA-exposed animals is otherwise intact.

### Baseline Conditions

**Figure 1:**
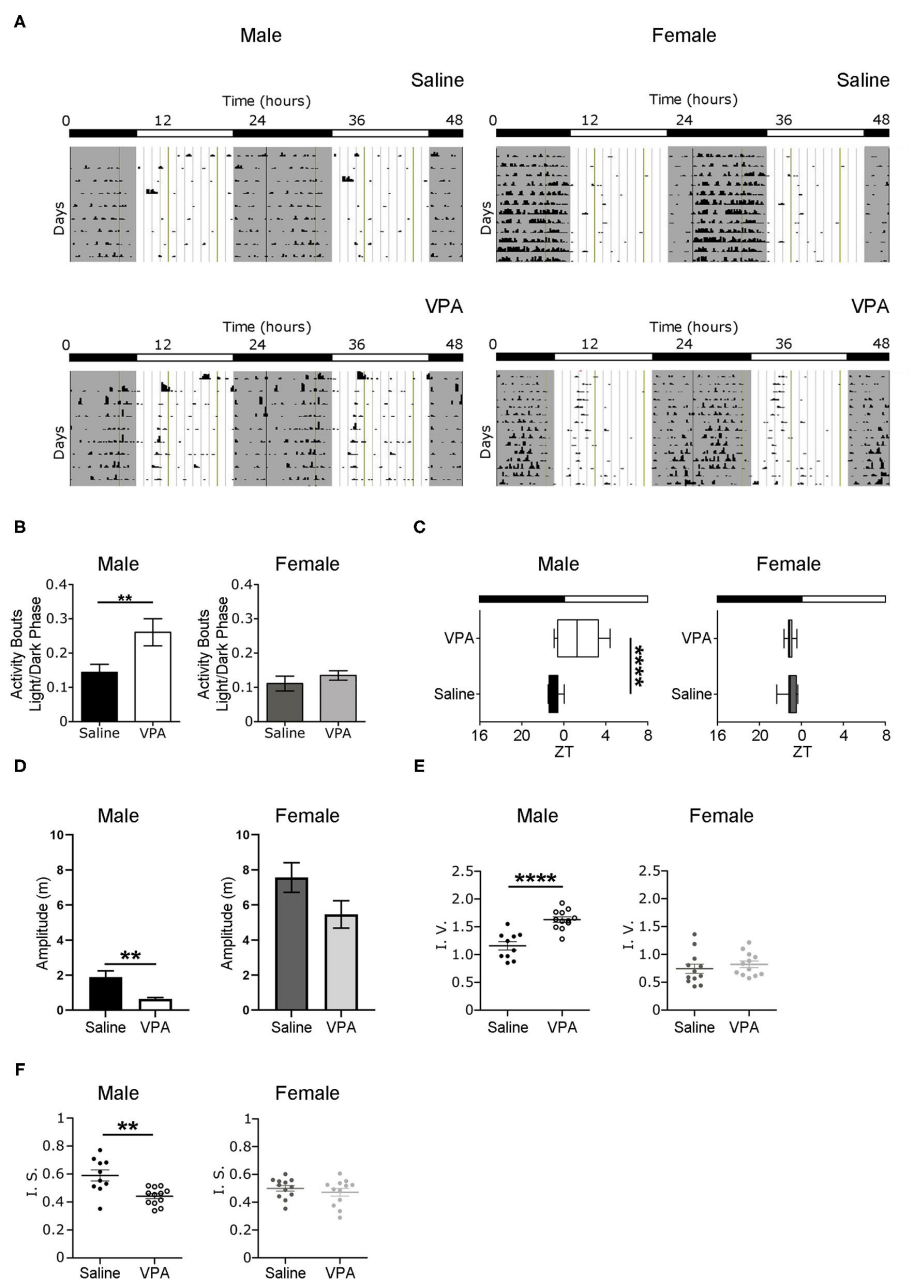
*In utero* VPA exposure alters wheel-running behaviour under baseline conditions. **(A)** Representative double-plotted actograms from (left panel) saline-exposed males (*n* = 10) and VPA-exposed males (*n* = 12). Right panels represent double-plotted actograms from saline-exposed females (*n* = 12) and VPA-exposed females (*n* = 12), maintained under 12-h light/dark conditions. Grey shaded areas indicate lights off [Zeitgeber time (ZT) 0 = lights on, ZT 12 = lights off]. **(B)** VPA-exposed males, but not females, show less circadian variation in wheel-running activity under baseline conditions (saline: M = 0.1438 ± 0.02346, VPA: M = 0.2607 ± 0.03958, *p* = 0.0256). Graphs depict a ratio of activity bouts during the light phase/ activity bouts during the dark phase (bout = 1 count of activity/minute/10 min). **(C)** Activity offsets in VPA-exposed males occur ~1.43 h into the light phase (saline, M= 7.062 ± 0.1655, VPA: M = 9.433 ± 0.5861 *p* = 0.0015, where 7:00 h corresponds to ZT 1). **(D)** Amplitude (m) is diminished in VPA-exposed males in comparison to controls, but not in VPA-exposed females (saline: M = 1.885 ± 0.3610, VPA: M = 0.6412 ± 0.07913, *p* = 0.0015). **(E)** VPA-exposed males show fragmented daily activity rhythms (intra-daily variability) (saline: M = 1.157 ± 0.07543, VPA: M = 1.631 ± 0.1735). **(F)** Inter-daily stability is decreased in VPA-exposed males (saline: M = 0.5899 ± 0.03950, VPA: M = 0.4413 ± 0.01779) indicating decreased photic-entrainment capacity. Data was analysed using a two-tailed unpaired *t*-test (or Welch's correction when appropriate) and are plotted as mean ± SEM. **p ≤ 0.01; ****p ≤ 0.0001.

Non-image forming pathways and circadian timekeeping are important for the generation of rhythmic behaviours but are equally integral in emotion regulation and metabolic processes; all of which are altered to some degree in ASD individuals (Karatsoreos et al., 2011; Legates et al., 2012). Therefore, we sought to determine whether *in utero* VPA-exposure altered circadian rhythmicity and if so, the extent of such alterations. To address whether VPA-exposed animals display circadian disturbances, we first examined wheel-running behaviour in rats housed under standard 12 h LD conditions. Under baseline conditions, VPA-exposed males displayed an inability to confine wheel-running activity to the dark phase—behaviour typical of nocturnal animals (Figure 1A). Rather, VPA-exposed males increased their ratio of day/night activity events, showing less diurnal variation in comparison to controls (*p* = 0.0256) (Figure 1B). As a result, the duration of the active phase (i.e., alpha) in VPA-exposed males was significantly extended by 2.2 h (*p* = 0.0149) (Supplementary Figure 2B) and resulted in activity offsets occurring ?1.43 h into the light phase (*p* = 0.0015) (Figure 1C). Importantly, VPA-exposed males did not demonstrate an increase in the number of overall activity events across 24 h, suggesting altered circadian distribution of wheel-running activity (Supplementary Figure 2C). Stable activity onsets and offsets are considered a marker of normal circadian functioning, while variability in this parameter is an indicator of circadian disruption. VPA-exposed males showed increased variability in their activity onsets in comparison to controls, indicating rhythm instability (Supplementary Figure 2A). Moreover, the amplitude of the behavioural rhythm was significantly lower in VPA-males, indicating decreased strength of the internal clock output (*p* = 0.0073) (Figure 1D). Analysis of intra-daily variability revealed a significant increase in VPA-exposed males, suggesting increased fragmentation in daily rhythms (*p* < 0.0001) (Figure 1E). Furthermore, inter-daily stability—a marker of synchronisation strength between the internal rhythm and the external LD cycle—was decreased in VPA-exposed males, alluding to alterations in entrainment capacity (*p* = 0.0047) (Figure 1F). Notably, these circadian alterations were not seen in VPA-exposed females, suggesting sexually dimorphic behaviour in circadian rhythmicity post-*in utero* treatment under baseline conditions. Collectively, these results suggest marked rhythm instability and dysregulation of the SCN clock output under baseline conditions in VPA-exposed males but not in VPA-exposed females.

### Dysregulation Under Constant Conditions

**Figure 2:**
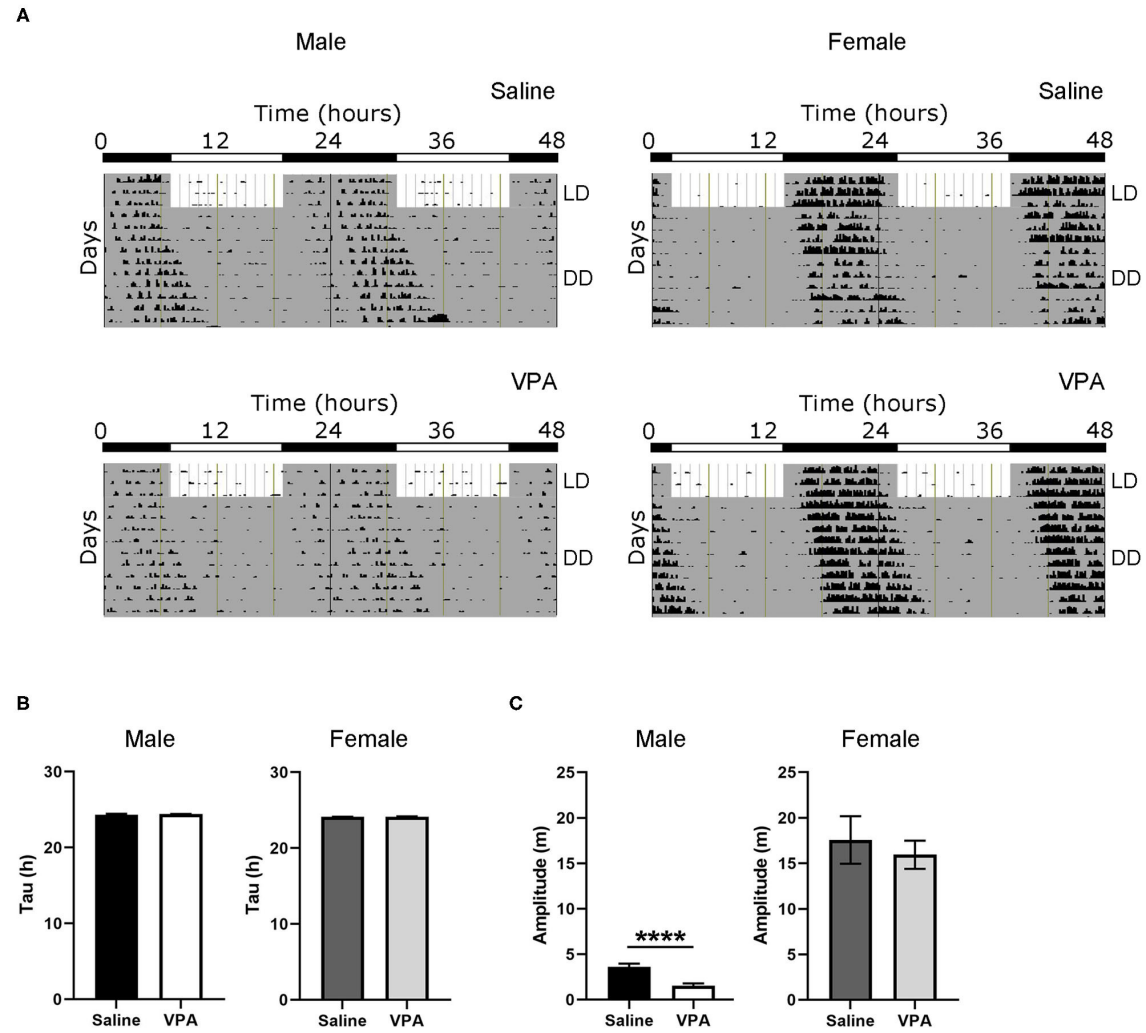
Behavioural rhythms are diminished in VPA-exposed males under constant dark (DD) conditions. **(A)** Representative double-plotted actograms from (left panel) saline-exposed males and VPA-exposed males. Right panels represent double-plotted actograms from saline-exposed females and VPA-exposed females, maintained under constant dark conditions. **(B)** Tau (hours), or the period of the endogenous free-running rhythm, is unchanged between VPA-exposed males/females and saline-exposed males/females. **(C)** Amplitude (m) is lessened under DD conditions in VPA-exposed males (saline: M = 3.618 ± 0.3464, VPA: M = 1.545 ± 0.8215, *p* < 0.0001). Data was analysed using a two-tailed unpaired *t*-test (or Welch's correction when appropriate) and are plotted as mean ± SEM. ****p ≤ 0.0001.

To further understand the circadian disturbances seen under baseline conditions in VPA-exposed animals, we examined the effects of constant conditions [constant dark (DD) and constant light (LL)] on rhythmicity (Figure 2A). Under DD conditions, neither males nor females displayed differences in their intrinsic free-running period (Figure 2B), suggesting that the observed circadian disturbances are not due to a lengthening or shortening of the molecular clock. Furthermore, alpha was unchanged between groups under DD conditions (Supplementary Figure 3B). This finding suggests that under baseline conditions, rhythm instability occurs through aberrant photic entrainment, consequently driving an increase in alpha. However, the amplitude of the intrinsic rhythm in DD was lowered in VPA-exposed males (*p* < 0.0001), suggesting decreased strength of the SCN clock output independent of light-induced effects (Figure 2C). As observed under baseline conditions, females exposed to VPA did not show any differences under DD conditions when compared to controls.

**Figure 3:**
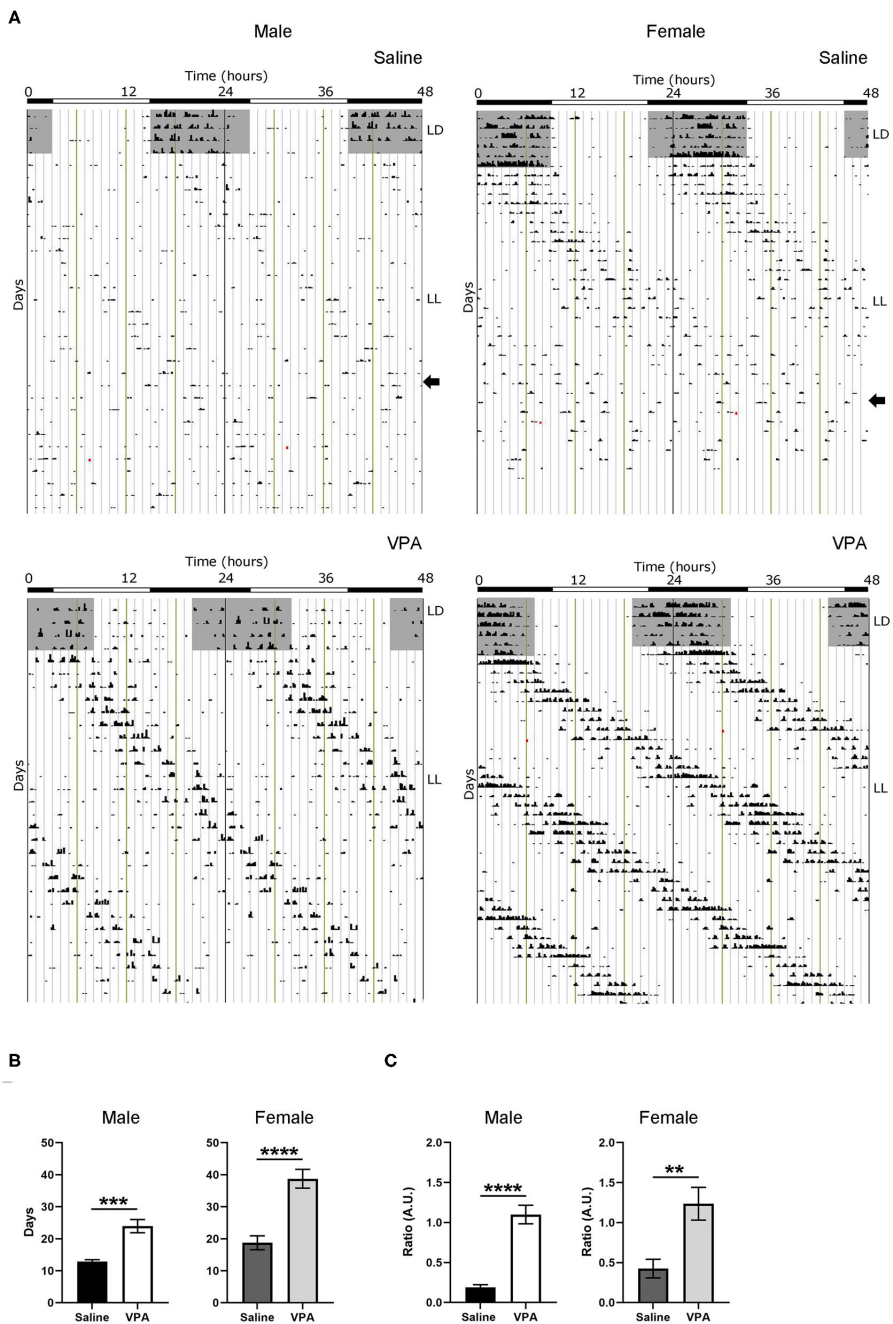
*In utero* VPA exposure alters locomotor suppression under constant light (LL) conditions in both sexes. **(A)** Representative double-plotted actograms from (left panel) saline-exposed males and VPA-exposed males. Right panels represent double-plotted actograms from saline-exposed females and VPA-exposed females, maintained under constant light conditions. **(B)** Both VPA-exposed males and females demonstrate an increase in the number of days needed to reach arrhythmic behaviour, in comparison to their respective control counter parts (males; saline: M = 12.89 ± 0.5638, VPA: M = 23.93 ± 2.069, *p* < 0.0001. Females: saline: M = 18.75 ± 2.171, VPA: M = 38.75 ± 2.926, *p* < 0.0001). **(C)** Both VPA-exposed males and females show an attenuated negative masking response under LL conditions, represented by a ratio of (10 d average of total activity in LL)/(10 d total activity in LD) (Males; saline: M = 0.1895 ± 0.03219, VPA: M = 1.100 ± 0.1169, *p* < 0.0001. Females; saline: M = 0.4248 ± 0.1167, VPA: M = 1.236 ± 0.2055 *p* = 0.0024). Data was analysed using a two-tailed unpaired *t*-test (or Welch's correction when appropriate) and are plotted as mean ± SEM. **p ≤ 0.01, ***p ≤ 0.001, ****p ≤ 0.0001.

Negative masking, or a decrease in locomotor activity in response to light, is commonly observed in response to constant light conditions in nocturnal animals (Mrosovsky and Hattar, 2003) (Figure 3A). However, under LL both males (*p* < 0.0001) and females (*p* = 0.0024) exposed to VPA demonstrated an attenuated negative masking response (Figure 3C). The LL paradigm also elicits loss of rhythmicity after prolonged exposure—an effect believed to occur due to uncoupling of SCN neurons (Ohta et al., 2005). Strikingly, in both VPA-exposed males (*p* < 0.0001) and females (*p* < 0.0001), the number of days needed to achieve arrhythmicity was significantly longer compared to controls, again suggesting an underlying impairment of photic integration in these animals (Figure 3B). Notably, while females did not display circadian alterations under baseline conditions, the results obtained during LL demonstrate circadian dysregulation under challenge conditions.

### Impaired Response to Phase-Shifting Light Pulse

**Figure 4:**
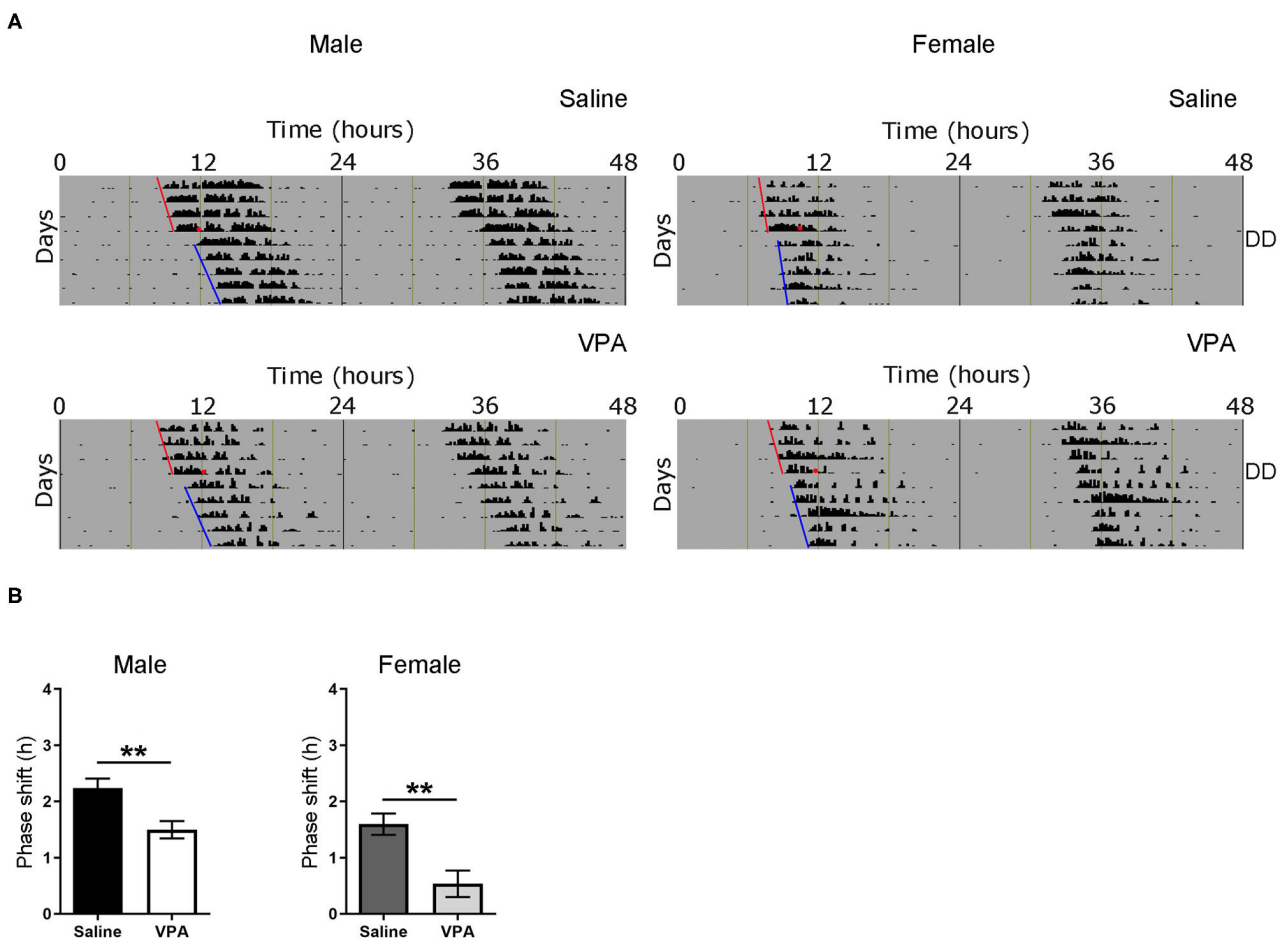
Altered responses to an Aschoff type-1 light pulse in VPA-exposed animals. **(A)** Representative double-plotted actograms from (left panel) saline-exposed males and VPA-exposed males. Right panels represent double-plotted actograms from saline-exposed females and VPA-exposed females, maintained under DD conditions for 6 days before a 1 h light pulse at CT 15 (3 h after the onset of wheel-running activity) was elicited (represented by the black circle). The subsequent phase shift in activity can be observed. **(B)** The averaged phase shift (h) over 5 days, for VPA and saline-exposed males/females. Both VPA-exposed male/female animals demonstrate a lessened phase-shifting response (Males; saline: M = 2.238 ± 0.1688, VPA: M = 1.495 ± 0.1549, *p* = 0.0043. Females; saline: M = 1.595 ± 0.1900, VPA: M = 0.5351 ± 0.2368, *p* = 0.0044). Data was analysed using a two-tailed unpaired *t*-test and are plotted as mean ± SEM. **p ≤ 0.01.

Based on the results obtained from baseline and constant conditions, we sought to determine whether the integration of photic information was impaired in VPA-exposed animals. The administration of a light pulse (LP) during the early subjective night or late subjective night elicits a phase delay or advance, respectively. This phenomenon known as the phase-response curve to light (PRC) can be utilised to assess plasticity of the SCN. Both saline and VPA-exposed males and females were released into DD conditions for 6 days before a 1-h, 150 lux LP was employed 3 h after the onset of running-wheel activity (Circadian Time, CT 15. Aschoff type 1 protocol) (Figure 4A). The subsequent phase shift in activity, measured over a 5-day period, was obtained for both groups (Supplementary Figures 3A,B). When exposed to light at CT 15, VPA-treated males displayed an attenuated phase-shifting response when compared to controls (*p* = 0.0043) (Figure 4B). Interestingly, similar observations were seen in VPA-exposed females when compared to controls (*p* = 0.0044). These results indicate that VPA-exposed animals display altered plasticity of the SCN in response to a phase-shifting light pulse, further suggesting aberrant photic-entrainment in these animals.

### Circadian Dysregulation in VPA-Animals Occurs Independently of Retinal Dysfunction

**Figure 5:**
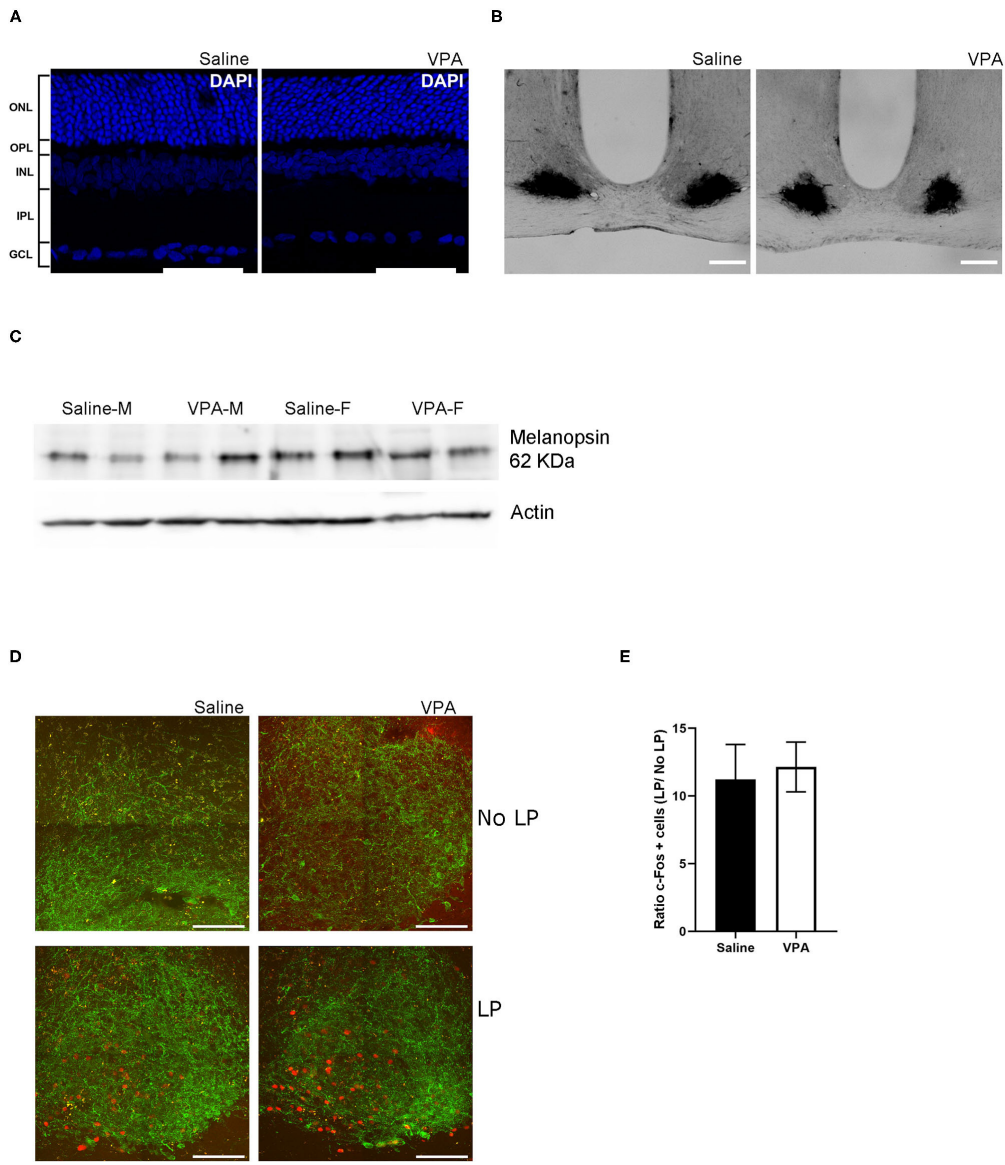
*In utero* VPA exposure does not alter retinal organisation. **(A)** Immunofluorescent staining for the nuclear marker, DAPI in saline and VPA-exposed animals. Note the normal organisation of all five layers of the retina [outer nuclear layer (ONL), outer plexiform layer (OPL), inner nuclear layer (INL), inner plexiform layer (IPL) and ganglion cell layer (GCL)] Scale bar = 100 μm. **(B)** p75-NTR immunoreactivity in the SCN of saline (*n* = 3) and VPA (*n* = 3) exposed animals. **(C)** Western blot from retinal lysates from saline and VPA-exposed male/female animals collected between ZT1-3 under standard 12:12 conditions. Note the presence of melanopsin in all groups. **(D)** Immunofluorescent images of c-FOS (red) and VIP (green) in light pulse (LP) or no LP conditions. Scale bar = 100 μm. **(E)** Ratio of the number of c-FOS immunoreactive cells after 1 h LP/number of c-FOS immunoreactive cells after No-LP (student's *t*-test, ns).

One area potentially contributing to the observed phenotype may be due to impact of *in utero* VPA exposure on the development of the retina. It is currently unknown whether VPA affects normal development of the retina and/or if it affects retinal input to the SCN, which is necessary for normal entrainment to LD cycles. To address the question of retinal development, an immunofluorescent (IF) staining of the retinal cell layers was performed using the nuclear marker DAPI. IF staining of the retina revealed no overt effect of *in utero* VPA exposure on the presence and normal organisation of all five layers of the retina (Figure 5A).

Previous research has documented the expression of p75-neurotrophin receptor (p75NTR), a neurotrophic factor, in retinal afferents projecting to the SCN and within the retinorecipient core of the SCN itself (Kiss et al., 1993; Bina et al., 1997). Retinal neurotoxic agents such as monosodium glutamate [a primary disruptor of retinal ganglion cells (RGC)], when administered during the neonatal period, abolishes p75NTR immunoreactivity within the SCN (Beaulé and Amir, 2001). To gain further insight into the origins of circadian disturbances in VPA-animals, we investigated the presence of intact RGC projection to the SCN through IHC assay for p75NTR. Within the SCN, p75NTR immunoreactivity was found in both groups, indicating that a certain degree of connectivity between the retina and the SCN had been established despite exposure to VPA *in utero* (Figure 5B).

iPRGCs express the specialised photopigment melanopsin and depolarize in response to short-wavelength irradiation (Provencio et al., 1998; Berson et al., 2002; Hattar et al., 2006). Seminal studies have documented that melanopsin knockout animals (*Opn 4*^−/−^) display attenuated negative masking response (Mrosovsky and Hattar, 2003). A western-blot analysis was conducted to determine whether melanopsin expression was indeed present in the retinas of VPA-exposed animals (Figure 5C). The assay revealed the presence of melanopsin in both males and females exposed to saline or VPA *in utero* (*N* = 6), hinting that a lack of melanopsin within iPRGCs likely does not underlie the attenuated negative masking response observed in VPA-exposed animals.

Expression of the immediate-early gene *c-fos*, can be used as a marker of neuronal activation in the SCN in response to light exposure during the early subjective night (Kornhauser et al., 1990, 1996; Rusak et al., 1990). To gain insight into whether light elicits the same neuronal response within the SCN, a 1-h, 150 lux LP at CT 15 was performed. Animals were sacrificed 1-h post LP and tissue was sectioned to obtain the SCN, followed by IF staining for c-FOS and vasoactive intestinal peptide (VIP) within the SCN (Figure 5D). Similarly, a no-LP condition was conducted to ensure that the expression of c-FOS after the 1-h LP condition did indeed exceed basal levels. No significant differences in the ratio of c-FOS positive cells in the SCN of either VPA or saline-treated animals were detected, suggesting that photic information was properly relayed from the retina to the SCN, and the same local response was elicited, highlighting potential downstream perturbations (Figure 5E).

Immunoreactivity for VIP was used to distinguish the ventral “core” of the SCN; however, VIP is equally important for the coupling of cellular oscillators and proper entrainment (Aton et al., 2005). Mice that lack VIP display weak behavioural rhythms, thus marked changes in VIP expression may contribute to the circadian disturbances in seen in VPA animals (Colwell et al., 2003; Brown et al., 2007). While we did not directly quantify VIP expression within the SCN, its expression pattern from the ventral core towards the dorsal shell did not appear to differ between groups in mid-coronal SCN slices. This may suggest that changes in VIP expression are unlikely to contribute to circadian alterations in our model (Supplementary Figure 4). Collectively, the results from these assays highlight that circadian dysregulation in VPA-treated animals occurs independently of retinal dysfunction.

### BMAL1 Expression Within the SCN of VPA-Exposed Animals

**Figure 6:**
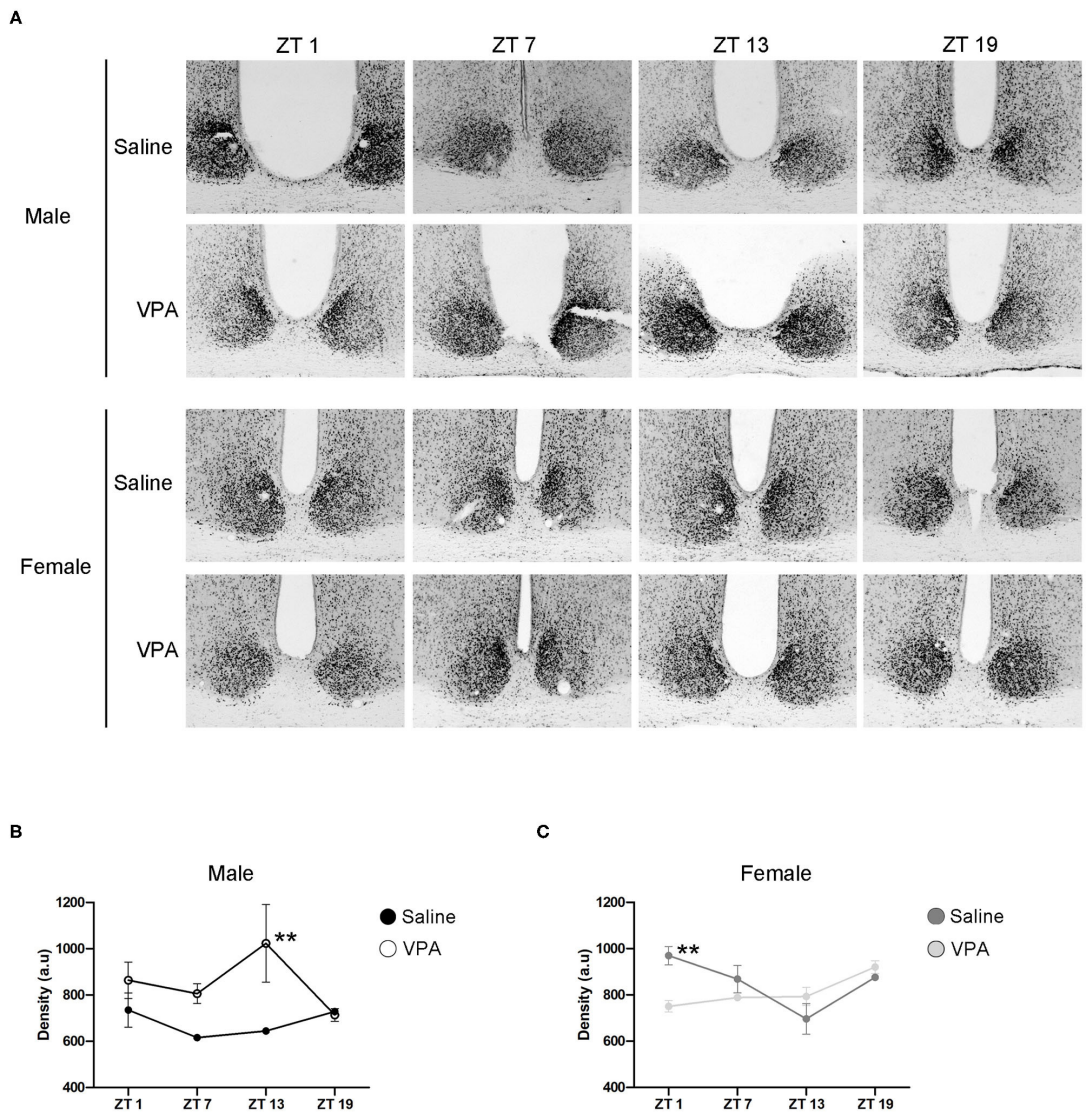
*In utero* exposure to VPA alters the expression of BMAL1 within the SCN. **(A)** Representative images of BMAL1 immunoreactivity within the SCN of saline and VPA-exposed males (upper panels) and females (lower panels) across a 24 h period (ZT 1, 7, 13, 19), where *n* = 3/time point/condition in both sexes. **(B)** Differential BMAL1 expression within the SCN of VPA-exposed males [two-way ANOVA, significant main effect of treatment. *F*_(1, 14)_ = 15.10, *p* = 0.0016]. Planned comparisons revealed a significant difference at ZT 13 between groups (*p* = 0.0044). **(C)** BMAL1 expression within the SCN of VPA-exposed females is altered compared to saline-exposed females [two-way ANOVA, significant interaction of treatment × time. *F*_(3, 16)_ = 6.227, *p* = 0.0053]. Planned comparisons revealed a significant difference in BMAL1 expression at ZT 1 between groups (*p* = 0.0053). Data was analysed using a two-way ANOVA and a Bonferroni planned comparison of time points. **p ≤ 0.01.

Circadian timekeeping within the master clock is achieved through the rhythmic expression of clock-genes within neurons of the SCN (Takahashi, 2017). Global or region-specific loss of the clock-gene *Bmal1* has been shown elicit a loss of behavioural rhythms and negatively affects overall physiological functioning (Bunger et al., 2000; Kennaway et al., 2013; Sutton et al., 2017). As such, BMAL1 positions itself as a necessary component of the core molecular clock. To gain mechanistic insight into our behavioural phenotype, we characterised the expression of BMAL1 at four time points over a 24 h period within the SCN under standard conditions (Figure 6A). In VPA-exposed males, BMAL1 followed an abnormal expression pattern compared to controls [main effect of treatment, *F*_(1, 14)_ = 15.10, *p* = 0.0016], peaking at the beginning of the dark phase (ZT 13), whereas BMAL1 expression in controls did not demonstrate any observable rhythmicity (Figure 6B). Moreover, elevated expression of BMAL1 was detected in VPA-exposed males relative to their control counterparts. Surprisingly, while VPA-exposed females did not show circadian dysregulation under baseline conditions, BMAL1 also demonstrated abnormal expression in comparison to controls [significant interaction, *F*_(3, 16)_ = 6.227, *p* = 0.0053] (Figure 6C). These results highlight that altered behavioural rhythmicity in VPA-exposed animals is reproduced by alterations at the level of the molecular clock and is reflective of desynchronization between external zeitgebers and clock-gene expression.

### VPA Animals Display Altered Temporal Profiles of CORT

**Figure 7:**
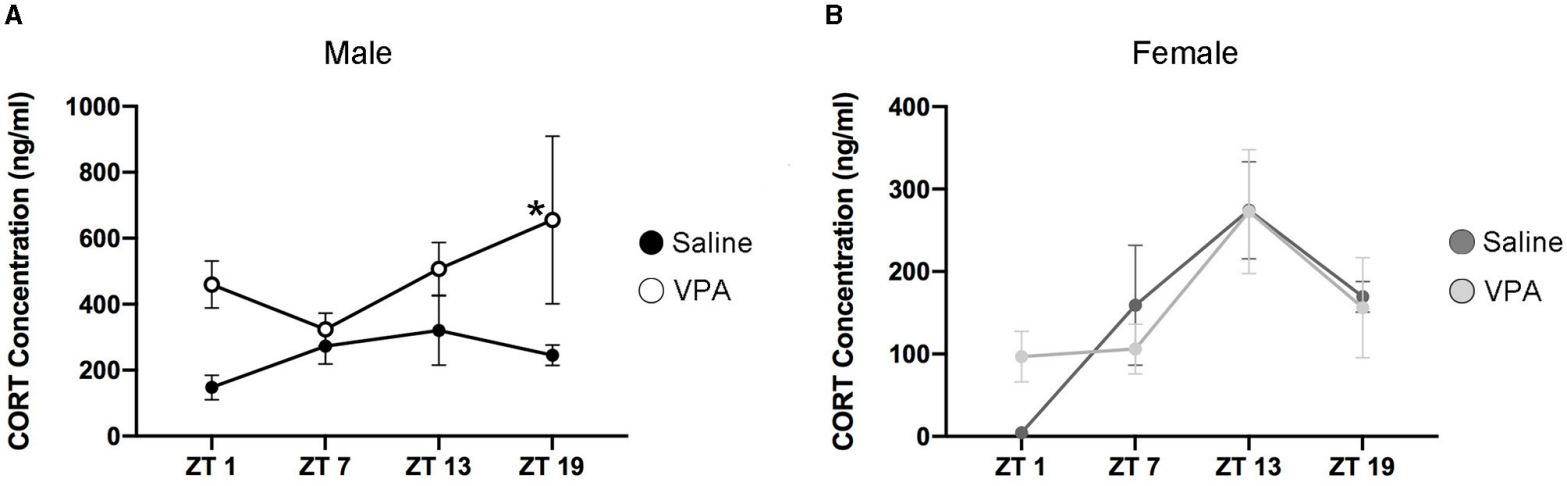
Altered temporal secretion profiles of CORT in VPA-exposed animals. **(A)** Plasma CORT levels for saline and VPA-exposed males and **(B)** females, under standard 12:12 LD conditions, collected over a 24 h period (ZT 1, 7, 13, 19, *n* = 2–3/time-point). *In utero* exposure to VPA alters secretion of CORT (ng/ml) in males, but not females [two-way ANOVA, significant main effect of treatment, *F*_(1, 13)_ = 14.35, *p* = 0.0023]. Planned comparisons revealed a significant difference in CORT secretion at ZT 19 between groups (saline: M = 1,251, VPA: M = 3,281, *p* = 0.0321). Means ± SEM are shown. Data was analysed using a two-way ANOVA and a Bonferroni planned comparison of time points. * ≤ 0.05.

Cortisol in humans and corticosterone (CORT) in rodents display distinct temporal secretion profiles that are largely under the control of the master clock (Spiga et al., 2014). Cortisol secretion in humans' peaks in the morning hours shortly after awakening, declines rapidly over the course of the morning and finally decreases to its lowest point during the evening. However, in rodents the peak of CORT secretion occurs near the onset of the active phase. Since VPA-exposed males display circadian disturbances at both the behavioural and molecular level, we sought to determine whether downstream physiological functioning under the control of the SCN clock was also disturbed. An enzyme-linked immunosorbent assay (ELISA) was conducted for CORT in plasma samples taken at four timepoints over 24 h. Similar to the results obtained from BMAL1 expression, CORT secretion in VPA-exposed males showed a marked difference in its daily secretion profiles [main effect of treatment, *F*_(1, 13)_ = 14.35, *p* = 0.0023] and appears to be elevated (Figure 7A). However, in VPA-exposed females, no significant differences in secretion level, or temporal profiles were detected, despite the observed alterations within the core clockwork (Figure 7B).

## Discussion

Here, we have provided several lines of evidence that demonstrate that the VPA-induced animal model of autism spectrum disorder shows circadian dysregulation of behaviour and physiology, likely through aberrant photic entrainment. We found that VPA-exposed males, but not females, demonstrate unstable and diminished output from the central circadian clock under baseline conditions. However, under constant conditions (DD) we observe no changes in the free-running period of these animals, albeit that the output strength of the clock remains lessened. These results suggest that alterations in these animals are driven, in part, through abnormal integration of photic information. To further this point, under constant light conditions (LL) we observe, in both males and females exposed to VPA, an increase in the number of days needed to achieve arrhythmicity and an attenuated negative masking response. In response to an Aschoff type-1 light pulse, VPA-exposed animals also demonstrate an attenuated phase-shifting response when compared to controls. Taken together, these results suggest the existence of a diminished central circadian oscillator in VPA-exposed animals, in which decreased photic entrainment capacity further drives instability of behavioural rhythms under baseline and LL conditions.

We have demonstrated that circadian alterations in VPA-animals occur independently of retinal dysfunction. Exposure to VPA *in utero* has no effect on the organisation of retinal layers, nor on the gross integrity of the retinohypothalamic tract, responsible for relaying photic information from the retina to the SCN (Berson et al., 2002). Melanopsin, a specialised photopigment that aids in the depolarization of retinal ganglion cells in response to short-wavelength irradiation (Hattar et al., 2006), was found to be present in VPA-exposed animals and is therefore unlikely to contribute in any significant way to the observed abnormal behavioural phenotype. We have also reported that local neuronal responses, examined through c-FOS expression within the SCN in response to a 1-h LP at CT 15, did not differ between groups. Collectively, these data suggest that retinal dysfunction, either at the level of the retina itself or through its connexion to the SCN, does not drive circadian alterations in VPA-animals. Moreover, since localised responses to the 1-h LP in VPA animals did not differ between groups, we propose a downstream mechanism to be responsible for the disturbances seen in these animals.

Input pathways that allow environmental stimuli to reset the clock, the master pacemaker, and output pathways that regulate physiological and behavioural processes are the necessary components that govern circadian rhythmicity. Global loss of *Bmal1* elicits an immediate loss of rhythmic behaviours in DD and decreased entrainment capacity under 12 h:12 h conditions, indicating that *Bmal1* is necessary for the generation and maintenance of normal circadian behaviour (Bunger et al., 2000). Although it has been well documented that mRNA levels of *Bmal1* oscillate within the SCN, there has been much less consensus with regards to the rhythmicity of its cognate protein. Here, we show constitutive expression of BMAL1 in control animals, consistent with the previously reported findings of others (Gall et al., 2003; Wyse and Coogan, 2010). Intriguingly, BMAL1 not only showed markedly different expression profiles in VPA-exposed males in comparison to saline-exposed males, but also appeared to be increased. While we have determined that the input pathway to the SCN remains intact, these findings suggest alterations within the core-clockwork itself. However, it is still unknown how other members of the canonical clock are affected after *in utero* exposure to VPA and warrants future investigation. Moreover, whether alterations within the core TTFL represent a direct mechanistic cause of altered circadian behaviour, or whether these alterations arise as a secondary consequence of *in utero* VPA exposure is discussed in detail below.

SCN output signals are diverse, including humoral output and neuronal projections emanating from the SCN to distinct hypothalamic and thalamic regions (Inouye and Kawamura, 1979; Silver et al., 1996; Saper et al., 2005). However, the full mechanistic extent by which the SCN exerts its control over downstream physiological rhythmicity is not entirely known. One key output of the master clock is the cyclical nature of the spontaneous firing rate of SCN neuronal ensembles (SFR), which encodes solar time through increased neuronal firing during the circadian day, and decreased activity during the circadian night. Downstream rhythmic behaviour is thought to be driven by the SCN's SFR, since behavioural cycles are tightly linked to rhythmic changes in SFR (Houben et al., 2014). However, changes to the SFR are also known to reset the phase of the TTFL, highlighting the reciprocal nature of input/output influences on rhythmicity of the molecular clockwork (Jones et al., 2015). Whether alterations in the SFR of VPA exposed animals are present and contribute to both the disturbances in BMAL1 expression at the level of the SCN and to the emergence of disorganised circadian behaviour is currently unknown. Additionally, VPA has been shown to alter neuronal excitatory/inhibitory balances, increasing glutamatergic tone, and decreasing GABAergic neuronal development (Fukuchi et al., 2009; Kim et al., 2014). Communication between neurons within the SCN is largely GABAergic (Moore and Speh, 1993), thus a shift in the excitatory/inhibitory balance within the SCN may also contribute to alterations in rhythmic behaviour and clock-gene expression, although this remains to be investigated.

While cortisol secretion in humans' peaks shortly after wakening, peak cortisol secretion in children with ASD has been shown to drift later relative to the LD cycle and is often found in increased concentrations, particularly during the evening hours (Richdale and Prior, 1992; Corbett et al., 2008). Elevated trough concentrations of cortisol have been associated with chronic stress, depression, decreased responsiveness of the hypothalamic-pituitary-adrenal (HPA) axis, and adverse metabolic consequences (Dallman et al., 2000). Corticosterone secretion—a downstream physiological marker under circadian control—is also altered in VPA-exposed males and resembles the abnormal cortisol secretion seen in ASD children, demonstrating a much later peak time relative to the LD cycle. Moreover, plasma CORT was found to be elevated especially in relation to the nadir of controls, suggesting alterations within the HPA-axis in these animals. While the HPA-axis is involved in stress responses in mammals, crosstalk between the HPA axis and the circadian clock has been well-documented (Rao and Androulakis, 2019). In part, the SCN exerts circadian control of glucocorticoid (GC) secretion by relaying photic information directly to the hypothalamic paraventricular nucleus, a region important in the GC secretion cascade (Kalsbeek et al., 2012). However, to achieve a state of internal synchrony, appropriate entrainment between the SCN and peripheral tissues is thought to occur partially through GC rhythmicity. Robust clock-gene expression in peripheral tissues is driven in part by rhythmic GC secretion and is dependent on the presence of a functional GC-receptor, highlighting the bi-directional relationship between both systems (Balsalobre et al., 2000). Chronic circadian misalignment between the external light-dark cycle and the SCN and consequently, between the SCN and peripheral tissues, has been associated with a host of adverse metabolic outcomes and alterations in neuronal complexity in brain regions involved in higher-order functioning (Karatsoreos et al., 2011). Clearly, the interaction between both systems not only affects downstream physiological functions, but also the adaptive capacity to modulate physiology and behaviour in the face of stressful situations, all of which have been shown to be altered in ASD individuals. One key limitation in examining the expression of both BMAL1 within the SCN and CORT secretion is our inherently small sample size. In order to fully discern changes across 24 h, we included 4 timepoints occurring at 6 h intervals. While this decision did indeed decrease our sample size (*n* = 3/timepoint/condition), we believe it to be more informative, in that critical information may have been obfuscated had we limited our timepoints to morning vs. evening time.

ASD is found to occur at much higher rates in males than in females, although the biological origins of this discrepancy are still unclear. While there are many theories that aim to explain the presence of this sex bias, the proposition that many factors including risk genes and environmental insults, interact to increase the risk of ASD development in males, remains common to most hypotheses (Ferri et al., 2018). Recent findings within the field of ASD also delineate the complicated nature of studying sexually dimorphic behaviour in ASD, as it has been proposed that many females with ASD go undiagnosed due to a male-conceptualised model of ASD behaviour, thereby overestimating the male: female ASD ratio (Kreiser and White, 2014). Although understanding this sex bias in ASD is complicated by both biological and sociological factors, it remains an important factor in understanding the underlying biology of ASD. Moreover, there are known differences in circadian behaviour between the sexes both in humans and laboratory animals. Gonadal hormones influence the intrinsic free-running period in rats, but it is unknown if their effect is exerted at the level of the central clock, or on downstream regions which govern locomotor behaviour (Albers, 1981; Schull et al., 1989). Interestingly, research has shown decreased prevalence and severity of sleep-wake cycle alterations in females with ASD compared to their male counterparts (Sivertsen et al., 2012; May et al., 2015). However, one key limitation of these data is that females diagnosed with the disorder are often identified late in adolescence or adulthood, leading to a decreased sample size of females with confirmed ASD diagnoses in childhood, an age at which most subjects are selected for conducting sleep-wake cycle studies (May et al., 2015; Ferri et al., 2018). Here, we find that VPA-exposed females did not display abnormal behavioural rhythmicity under 12 h:12 h conditions but displayed overt circadian alterations under challenge conditions. Moreover, they demonstrated an attenuated phase-shifting response to an Aschoff-type 1 light pulse, indicating decreased plasticity of the central pacemaker. However, these results are particularly concerning; while our study was conducted in a controlled environment, chronic circadian challenges are common in today's industrialised world. It is possible that under perfect standard conditions, ASD females might display normal rhythmicity, but realistically, are at risk of circadian dysregulation and its impacts due to modern living challenges. While this remains a hypothesis, it is an important consideration for studies to come. Moreover, despite normal behavioural rhythmicity under 12 h conditions, the daily profile of BMAL1 expression within the SCN was abnormal relative to controls, further suggesting alterations within the master-clock itself in VPA-exposed females.

Characteristics of circadian disorders include difficulty awakening at an appropriate time, difficulty initiating and maintaining sleep, and decreased alertness during the day; all of which are common complaints in ASD individuals. Key challenges surrounding data collection in children with ASD and reliance on parental reports highlight the suitability of the VPA model, which allows for further understanding of the biological mechanisms that underlie alterations in these individuals. However, it is important to note the current limitations of this model; while it appropriately replicates the behavioural and molecular abnormalities associated with idiopathic ASD, most autistic individuals have not been exposed to this drug *in utero*, suggesting that findings from this model may be limited to ASD individuals who have been exposed to this class of medication during gestation. Nonetheless, there is consensus among experts that the aetiology of ASD is likely multifactorial in nature, and that these factors converge onto similar molecular pathways leading to the development of the disorder. Moreover, *in utero* exposure to VPA in humans results in a similar prevalence of ASD in both male and female offspring. However, a growing body of evidence suggests a sexually dimorphic presentation of the disorder between the sexes, in which females demonstrate fewer socio-communication difficulties compared to their male counterparts, but display increased internalising behaviours and elevated risk of comorbid affective disorders (Lai et al., 2011; Solomon et al., 2012). Indeed, research utilising the VPA rodent model has succeeded in revealing key differences in the emergence and expression of behavioural abnormalities between the sexes. In specific, VPA-exposed males show a marked decreased in social behaviours, including deficits seen in the olfactory discrimination task and social interaction task, which are not seen in VPA-exposed females. However, altered behaviour in the open field and elevated plus maze, markers of anxiety-like behaviours, as well as increased stereotypic behaviours have been observed in both males and females exposed to VPA *in utero*. These findings highlight that the VPA model effectively recapitulates the sexually dimorphic expression and emergence of ASD behaviours seen within human populations. Notably, we have demonstrated the existence of circadian alterations in our model highly reminiscent of sleep-wake cycle disturbances seen in ASD populations, further lending face validity to this animal model. Our results indicate that while input pathways remain intact and functional, the central pacemaker and possibly downstream output pathways are involved in the aetiology of circadian disturbances in this ASD model. Future experiments investigating the electrical output of SCN neuronal ensembles would help elucidate whether known alterations in the excitatory/inhibitory balance contributes to diminished and unstable output from the master clock. Our results and the necessity of future investigations are critical, as they may lead to new therapeutic approaches for treating circadian disorders concomitantly observed with a variety of neurodevelopmental disorders.

## Data Availability Statement

The raw data supporting the conclusions of this article will be made available by the authors, without undue reservation.

## Ethics Statement

The animal study was reviewed and approved by Animal Care Committee of Concordia University.

## Author Contributions

The experiments were conceived and designed by SF and SA. Experiments were conducted by SF and assisted by NdZ for experiments five, six, and seven. NB assisted with experiment five. Data was analysed and interpreted by SF with input from SA. Data visualization was done by NdZ and SF. Manuscript production was written by SF with editing by SA. SA supervised the project. All authors contributed to the article and approved the submitted version.

## Funding

This work was funded by a grant from the Canadian Institutes of Health Research (MOP142458) to SA.

## Conflict of Interest

The authors declare that the research was conducted in the absence of any commercial or financial relationships that could be construed as a potential conflict of interest.

## Publisher's Note

All claims expressed in this article are solely those of the authors and do not necessarily represent those of their affiliated organizations, or those of the publisher, the editors and the reviewers. Any product that may be evaluated in this article, or claim that may be made by its manufacturer, is not guaranteed or endorsed by the publisher.
