# Supplementary material for: In utero Exposure to Valproic-Acid Alters Circadian Organisation and Clock-Gene Expression: Implications for Autism Spectrum Disorders
Source: Front Behav Neurosci. 2021 Sep 28;15:711549. doi: 10.3389/fnbeh.2021.711549 (PMC8505722; doi:10.3389/fnbeh.2021.711549)
Supplement: Supplementary file 1 [file Image_1.pdf]

Supplemental Information

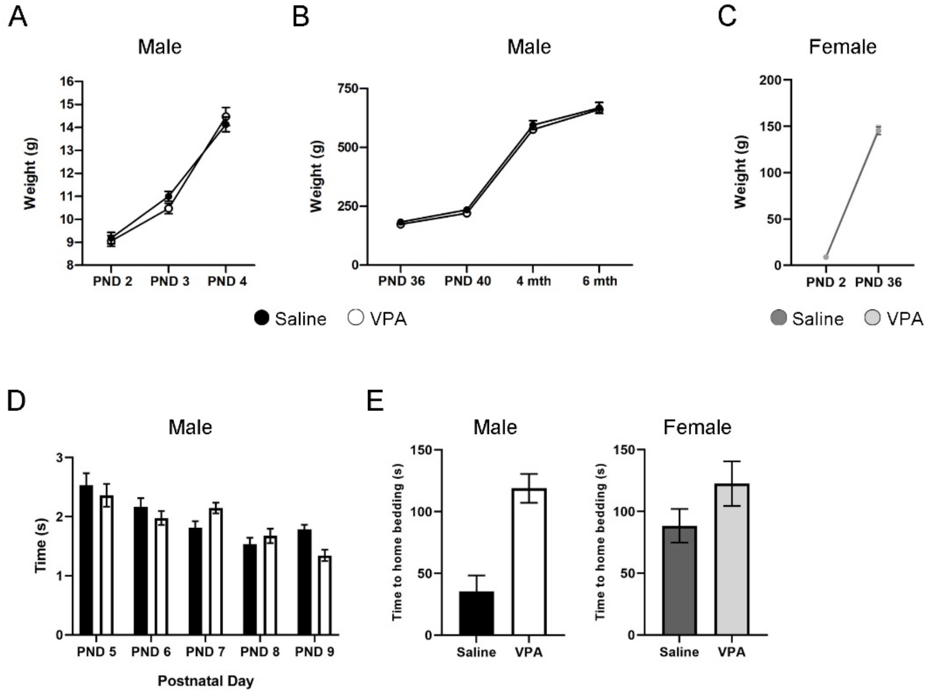


**Supplemental Figure 1. General development and health in saline and VPA-exposed animals.**

**(A)** Weight (g) of saline (n = 16) and VPA-exposed (n= 17) male pups shortly after birth on PND 2, 3 and 4 (two-way ANOVA, significant main effect of time, $F(2,91) = 175.4$, $p < 0.0001$). **(B)** Weight (g) of saline (n = 5) and VPA-exposed male pups (n = 6) after weaning and into adulthood (two-way ANOVA, significant main effect of time $F(3,36) = 761.4$, $p < 0.0001$). **(C)** Postnatal and post-weaned weight of saline (n = 13) and VPA (n = 24) exposed animals (two-way ANOVA, significant main effect of time, $F(1, 56) = 8269$, $p < 0.0001$). **(D)** Righting reflex task to assess motor development on PND 5, 6, 7, 8, 9 in saline and VPA exposed animals. Time to right themselves ventrally was recorded in seconds (two-way ANOVA, significant main effect of time, $F(4,284) = 13.02$, $p < 0.0001$). **(E)** Time to engage with home bedding (s) in saline (n = 18) and VPA (n = 13) exposed animals (saline: M = 35.50 ±12.86, VPA: M = 118.9 ±11.66, $p = 0.0005$). **(F)** Time to engage with home bedding (s) in saline (n = 10) and VPA (n = 31) exposed animals. No significant differences were detected. Means ± SEM are shown. Data was analyzed using a two-way ANOVA, or an unpaired two-tailed t-test.

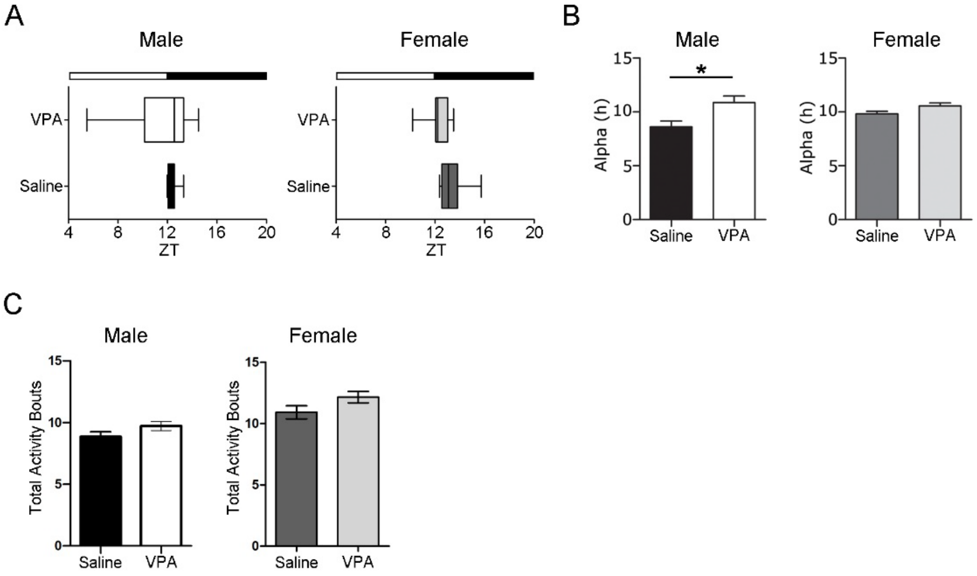


**Supplemental Figure 2. Behavioural rhythms are diminished in VPA-exposed males under constant dark (DD) conditions.**

**(A)** Onset (h) analysis for saline and VPA-exposed animals. **(B)** Alpha (h) is longer in VPA-exposed males under 12h:12h conditions (saline: M = 8.623 ±0.5374, VPA: M = 10.86 ±0.6222, $p = 0.0149$), but not in VPA-exposed females (right). Means ± SEM are shown. Data was analyzed using a one-way ANOVA, or an unpaired two-tailed t-test.

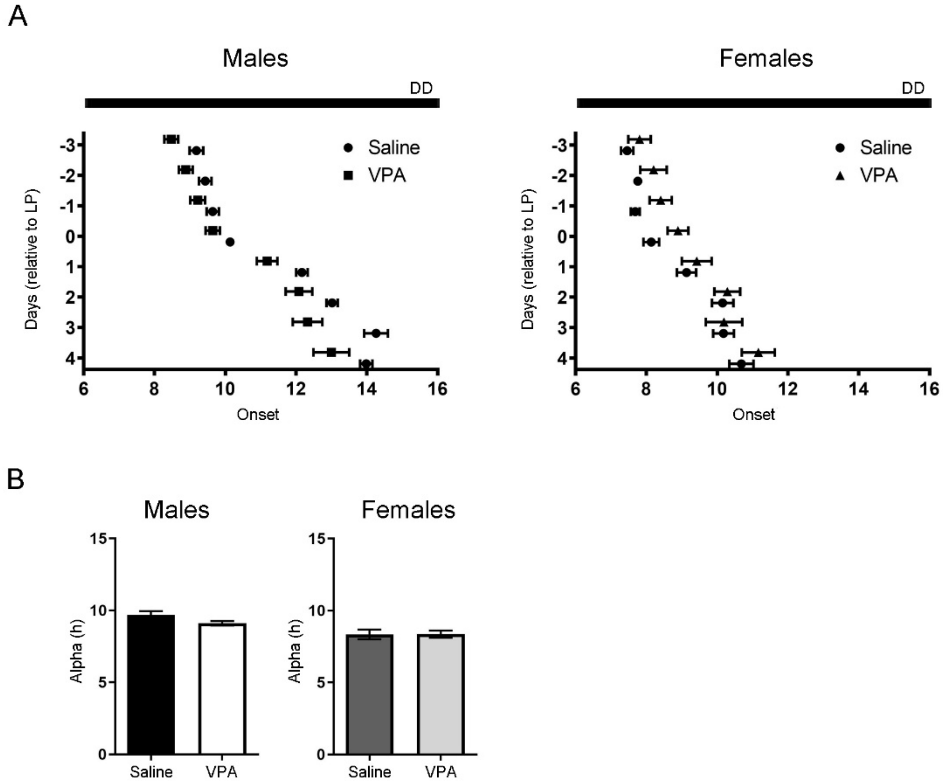


**Supplemental Figure 3. Altered responses to an Aschoff type-1 light pulse in VPA treated animals.**

**(A)** Group mean onsets prior to the light pulse given at CT 15 (denoted as day 0) and after. Note that VPA exposed males and females show an attenuated phase shifting response to the light pulse. **(B)** Alpha (h) in DD is not different between groups.

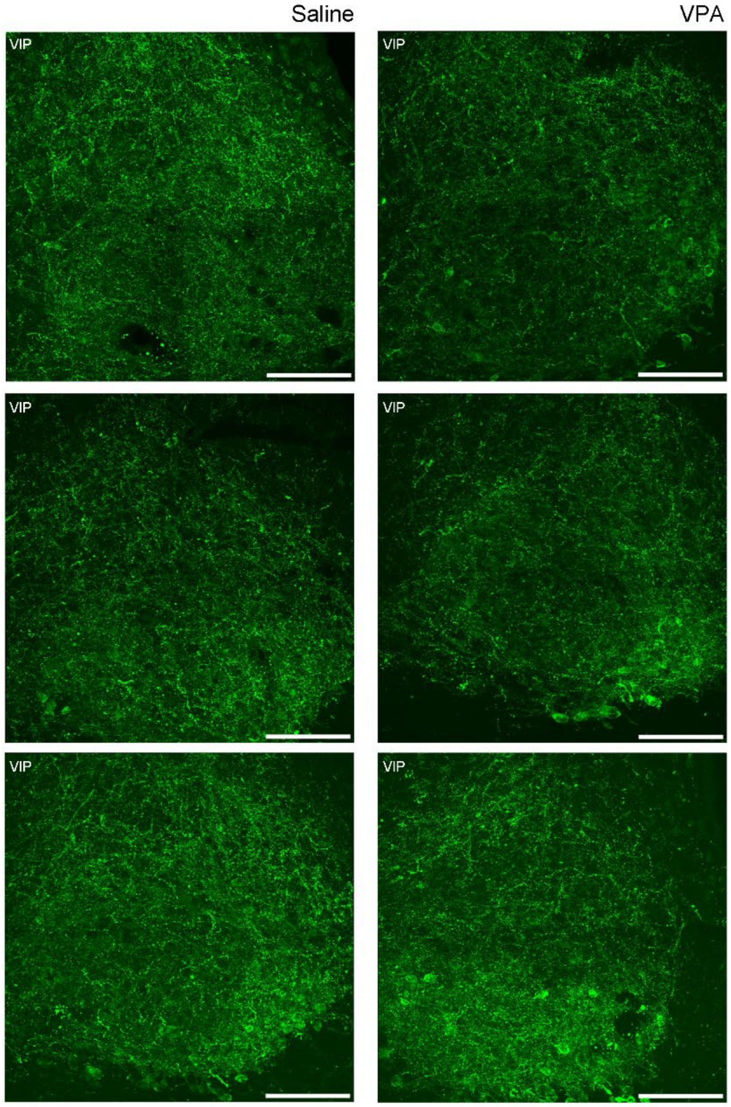


**Supplemental Figure 4. The distribution of a key neuropeptide, VIP, does not differ in the SCN of saline or VPA-exposed animals.**

**(A)** Mid-coronal SCN slices from saline (left panels) and VPA (right panels) exposed animals (60x). Green immunohistochemical staining represents with an anti-vasoactive intestinal polypeptide (VIP) immunoreactivity. Note that VIP is localized within the processes in the dorsal SCN.
